# Ultrasound‐Guided, Guidewire‐Assisted Long Peripheral Catheter in the Great Saphenous Vein for Bilateral Upper Limb Fracture Surgery: A Case Report

**DOI:** 10.1155/cria/8833164

**Published:** 2026-08-23

**Authors:** Mirco Leo, Benedetta Savarese, Gianmaria Cammarota

**Affiliations:** ^1^ IRCCS AOU SS Antonio e Biagio e Cesare Arrigo, Alessandria, Italy

**Keywords:** case report, difficult venous access, long peripheral catheter, perioperative care, saphenous vein

## Abstract

**Background:**

Establishing reliable venous access can be particularly challenging in trauma patients with bilateral upper limb fractures, where peripheral cannulation is unfeasible and central venous catheterization may be unnecessary or refused.

**Case Presentation:**

A 51‐year‐old woman (BMI 38 kg/m^2^) sustained bilateral elbow fractures requiring staged fixation. Because the patient expressed strong concern and reluctance toward central venous catheterization, and there was no clear indication for it, alternative options were explored. Ultrasound assessment revealed a suitable right great saphenous vein (GSV) with ≈ 3.6 mm of diameter. Under sterile and ultrasound‐guided conditions, a 10‐cm 18‐G PowerGlide long peripheral catheter (LPC) was successfully inserted, providing stable access for perioperative management over 10 days, with no complications or malfunction.

**Discussion:**

Midline and LPC are designed for intermediate‐term therapy and are typically placed in upper‐extremity veins. Their use in the saphenous vein for perioperative care has not been previously described. In this case, ultrasound‐guided saphenous LPC placement provided an alternative route for vascular access in a patient with extremely difficult venous access, without catheter‐related complications.

**Conclusion:**

Ultrasound‐guided placement of a 10‐cm LPC in the GSV provided perioperative venous access for 10 days without complications in this case. Further case series or prospective registries are needed to define patient selection, dwell time, and complication rates for lower‐extremity peripheral vascular access.

## 1. Introduction

Reliable intravenous access is crucial for safe perioperative management, particularly in trauma patients requiring staged orthopedic procedures [1, 2]. In patients with bilateral upper limb fractures, conventional peripheral cannulation is practically impossible.

The alternative options consisted of central venous catheterization via the internal jugular, subclavian, or femoral veins, or cannulation of the great saphenous vein (GSV) for a conventional venous catheter.

Before the surgical procedure, these alternative options in our case were made difficult by the patient’s BMI 38 kg/m^2^ and by her refusal and strong concern about undergoing such procedures. Furthermore, central venous catheterization would have represented a disproportionate intervention, as there was no strict indication for central access: The patient did not require administration of hyperosmolar or vesicant drugs, parenteral nutrition, or hemodynamic monitoring. Cannulation of the GSV for a conventional venous catheter was also considered technically challenging, if not impossible.

Ultrasound‐guided midline catheters are generally inserted into upper‐extremity veins for intermediate‐term therapy, and to the best of our knowledge, no reports describe their use in the GSV for perioperative management. We report a case in which a 10‐cm 18G PowerGlide long peripheral catheter (LPC) was successfully inserted into the right GSV, providing a vascular access throughout the perioperative period without the need for central venous catheterization. This case report was prepared in accordance with the CARE reporting guidelines.

## 2. Case Presentation

A 51‐year‐old woman (BMI = 38 kg/m^2^) with a history of hypertension under pharmacological treatment sustained bilateral elbow fractures after an accidental fall, without other traumatic injuries. The orthopedic team planned staged surgical fixation of both elbows, first on one side, and 6 days later, on the contralateral limb. On admission to the operating room, one upper limb was immobilized in a cast, while the other was scheduled for surgery. Because of the patient’s refusal and strong concern regarding central venous access, and the difficult palpation of lower‐extremity veins due to obesity, we decided to perform an ultrasound evaluation of the lower‐limb vasculature for potential vascular access.

**FIGURE 1:**
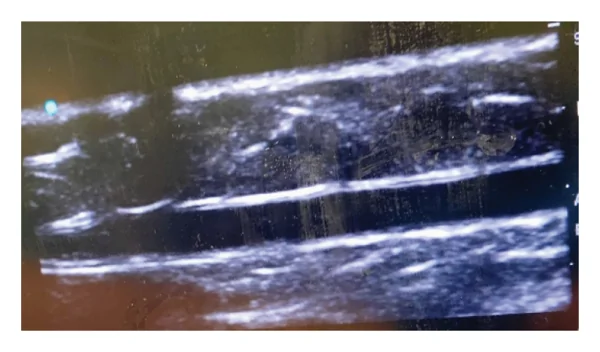
Ultrasound scan of the right great saphenous vein anterior to the medial malleolus.

**FIGURE 2:**
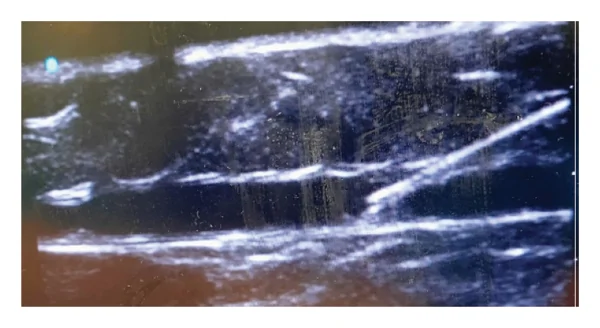
Ultrasound‐guided puncture of the right great saphenous vein.

**FIGURE 3:**
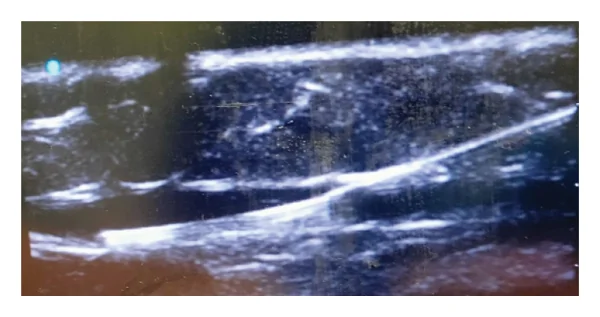
Insertion of the guidewire into the right great saphenous vein under ultrasound guidance.

**FIGURE 4:**
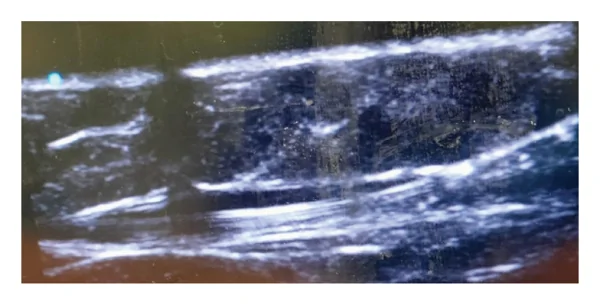
Insertion of the catheter over the guidewire.

**FIGURE 5:**
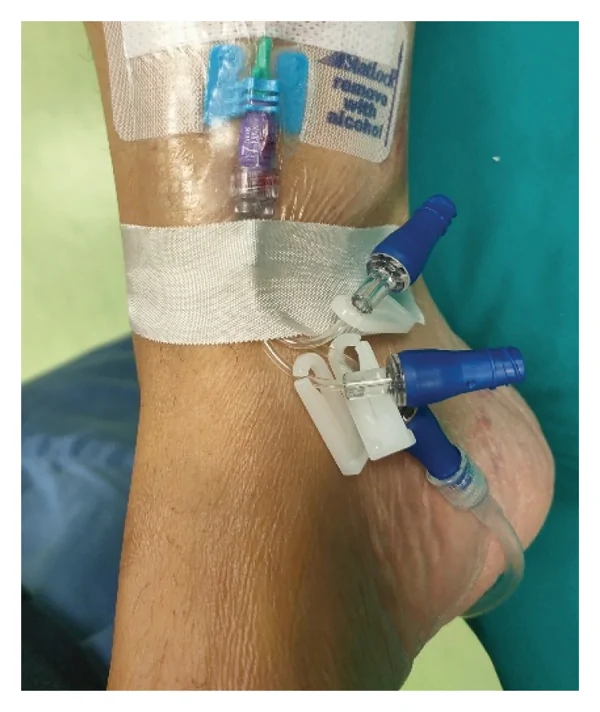
Securing and dressing of the vascular access site.

Before tourniquet placement, the right GSV was assessed for patency, compressibility, diameter, and suitability for catheter placement. The vessel appeared patent, fully compressible, and superficial on ultrasound despite not being clinically palpable, with an estimated diameter of approximately 3.6 mm (Figure 1). Although vein depth was not prospectively recorded as a numerical value at the time of cannulation, the vessel was judged suitably because it appeared superficial on ultrasound. Retrospective assessment of the saved ultrasound images, using the depth scale displayed on the ultrasound screen, showed that the vein was located approximately 1 cm from the skin surface. Assuming an approximate external diameter of 1.2‐1.3 mm for the 18‐G catheter used, the resulting catheter‐to‐vein ratio (CVR) would be approximately 35%. Given the 10‐cm catheter length and the superficial location of the vessel, the expected intravascular catheter length was considered adequate and likely well above the commonly recommended 65% threshold for catheter stability. On the basis of this preprocedural assessment, and after obtaining informed consent, we proceeded with ultrasound‐guided placement of a 10‐cm 18‐G PowerGlide LPC. The vessel was cannulated using an in‐plane ultrasound‐guided approach (Figure 2). After guidewire advancement (Figure 3), the catheter was advanced into position (Figure 4) and secured with a sterile transparent dressing (Figure 5). Based on the insertion site and catheter length, the tip was expected to remain within the GSV at approximately mid‐calf level, below the knee. The procedure was successful at the first attempt and was not associated with any periprocedural complications. The catheter was secured using the manufacturer‐supplied integrated securement device, consisting of an adhesive‐backed stabilization platform with a catheter‐locking mechanism, covered by a sterile transparent polyurethane dressing for daily inspection. No sutures or tissue adhesive were used. The midline was used for intravenous fluid administration and pharmacological therapy throughout the perioperative period. Infused fluids included 0.9% normal saline and lactated Ringer’s solution. Administered medications included antibiotics, corticosteroids, proton pump inhibitors, NSAIDs, and paracetamol. No parenteral nutrition, vasoactive drugs, vesicant or irritant medications, or infusates with extreme pH or osmolarity were administered. The catheter remained in place from the day of the first operation until discharge, 4 days after the second surgical procedure, for a total dwell time of 10 days. Catheter maintenance was performed according to institutional protocol, with flushing using 5–10 mL of 0.9% normal saline before and after each medication administration. When the catheter was not in active use (i.e., between the two surgical procedures), the lumen was locked with 0.9% normal saline using a positive‐pressure technique. No heparin lock was used. During catheter dwell time, follow‐up was performed according to routine clinical practice for peripheral and LPC vascular access in our department, based on daily clinical inspection of the insertion site and assessment of symptoms. No localized pain, erythema, palpable cord, swelling, warmth, pruritus, hyperpigmentation, induration, malfunction, or signs of infection were observed or reported by the patient. No Doppler ultrasound examination or vascular consultation was performed, as there were no clinical signs or symptoms suggestive of thrombosis or other vascular complications. No formal patient‐reported outcome measure or validated satisfaction questionnaire was collected. However, during postoperative follow‐up, the patient reported that she was satisfied with the proposed vascular access strategy, particularly because it avoided central venous catheterization, which had been a major source of concern for her.

## 3. Discussion

Midline catheters and LPC are intended for intermediate‐term intravenous therapy, typically lasting from 1 to 4 weeks (7–28 days), and are recommended when the anticipated duration of treatment exceeds that of a standard peripheral cannula but does not require central access. According to the Infusion Nurses Society (INS) Standards of Practice, 2024 (9^th^ edition), both provide a safe alternative to central venous catheters when infusates are compatible with peripheral administration, and central access is not otherwise indicated [2, 3]. Although the device used in this case was a LPC rather than a centrally inserted catheter (CIC), the insertion procedure adhered to general principles of systematic ultrasound‐guided vascular assessment that underpin established protocols such as the SIP/RaPeVA bundle for peripherally inserted central catheters (PICCs) and SIF/RaFeVA protocol for femorally inserted central catheters [4, 5].

Our case is distinguished by a combination of unfavorable factors: BMI 38 kg/m^2^, the patient’s refusal of central venous access, and the impossibility of obtaining conventional peripheral access in the upper limbs. Nevertheless, the catheter was used for intravenous fluid administration and pharmacological therapy throughout the perioperative period, with a total dwell time of 10 days and without catheter‐related complications. Several factors, however, should be considered when interpreting this outcome. First, apart from obesity, the patient was relatively young, had no significant comorbidities, was in good general condition, and was able to ambulate independently, although she required assistance with dressing and eating because of bilateral upper‐limb trauma. Therefore, the favorable outcome observed in this case may probably not be generalizable to older, frailer, bedridden, or multimorbid patients. Second, an important consideration when placing an LPC in the GSV is the potential alteration of lower limb hemodynamics. The lower extremity is inherently more susceptible to venous stasis due to gravitational hydrostatic pressure, which can reach up to 90mmHgin the standing position [6]. The presence of an intraluminal catheter reduces venous flow proportionally to the catheter CVR. In our patient, the CVR was approximately 35%. This value exceeds the 33% ratio as proposed by Bahl et al. for midline catheters but remains well below the 45% cutoff identified by Sharp et al. as the optimal threshold for predicting symptomatic venous thromboembolism in patients with PICC [7, 8]. We acknowledge that these thresholds have not been validated for LPCs in GSV. Third, the thrombotic risk associated with lower‐extremity vascular devices remains insufficiently characterized. In a prospective study evaluating ultrasound‐guided GSV cannulation in emergency department patients, Smoot et al. reported no clinically evident thrombosis, infection, or extravasation during 72 h of follow‐up. Nevertheless, the safety profile of lower‐extremity midline catheters beyond short‐term use remains unknown and warrants further investigation [9]. Fourth, lower‐extremity catheter placement may raise additional concerns regarding dressing stability, local contamination, infection risk, and interference with patient mobilization. In the present case, the patient was able to ambulate, the catheter site was regularly inspected, and no signs of infection, dislodgement, or catheter dysfunction were observed during the 10‐day dwell period. Nevertheless, these aspects should be carefully considered when selecting patients for lower‐extremity midline placement.

Finally, our experience should be interpreted within the broader context of lower‐extremity vascular access strategies. The search for reliable venous access in patients with limited or unavailable upper‐extremity options remains a significant clinical challenge. Lower‐extremity vascular access strategies have been described in selected settings, including the use of midline‐type catheters inserted in the superficial femoral vein at mid‐thigh [10]. This approach may represent a useful option in specific clinical scenarios; however, the femoral vein, despite its historical designation as the “superficial femoral vein,” is anatomically classified as a deep vein [11], and its cannulation should be considered within the broader risk profile of deep venous access, including catheter‐related thrombosis and infection [12].

In this context, the GSV represents a different anatomical option, as it is a superficial vein and may be accessible under ultrasound guidance in selected patients. Our case differs from both superficial femoral vein midline access and short‐term emergency department cannulation, as it describes the perioperative use of a 10‐cm LPC in the GSV when upper‐limb access was unavailable and central venous catheterization was unnecessary or declined.

Taken together, these experiences suggest that lower‐extremity LPC or midline‐type access may have a role in carefully selected patients. However, patient selection, ultrasound assessment, securement, maintenance, and monitoring for thrombosis or infection remain essential, and further comparative studies are needed to define the optimal strategy in this setting.

## 4. Conclusion

In this case, ultrasound‐guided placement of a 10‐cm 18G PowerGlide LPC in the GSV was uneventful and provided perioperative venous access for 10 days. Although no complications occurred, this single experience cannot support conclusions regarding safety or effectiveness. Case series and prospective registries are needed to better define the role, dwell time, and infectious, thrombotic, and overall complication profile of lower‐extremity peripheral vascular access.

## Funding

The article publication charge was covered by Becton, Dickinson and Company (BD).

## Conflicts of Interest

The authors declare no conflicts of interest.

## Supporting Information

Additional supporting information can be found online in the Supporting Information section.

## Supporting information


**Supporting Information 1** Supporting File 1: Patient timeline. Structured timeline summarizing the main clinical events from hospital admission to discharge and catheter removal.

**Supporting Information 2** CARE‐checklist saphenous midline catheter as a perioperative alternative to central venous access in bilateral upper limb trauma.

## Data Availability

Data sharing is not applicable to this article as no datasets were generated or analyzed during the current study.
