# Supplementary material for: Ultrasound‐Guided, Guidewire‐Assisted Long Peripheral Catheter in the Great Saphenous Vein for Bilateral Upper Limb Fracture Surgery: A Case Report
Source: Case Rep Anesthesiol. 2026 Aug 23;2026:8833164. doi: 10.1155/cria/8833164 (PMC13501016; doi:10.1155/cria/8833164)
Supplement: Supplementary file 2 [file CRIA-2026-8833164-s001.docx]

The timeline below summarizes the main clinical events of this case, from hospital admission for bilateral upper-limb fractures to discharge and catheter removal.

**Timeline**

| **Day 0** | Patient admitted to the Emergency Department after a fall resulting in bilateral upper-limb fractures requiring surgical treatment. |
| --- | --- |
| **Day 6** | Surgical fixation of the right elbow (radial head arthroplasty). During the perioperative period, an ultrasound-guided, guidewire-assisted 18G, 10-cm long peripheral catheter (PowerGlide™) was inserted into the right great saphenous vein. The catheter was used for perioperative drug administration, including proton pump inhibitors, corticosteroids, paracetamol, antibiotics, and NSAIDs. |
| **Day 12** | Surgical fixation of the left elbow with screw osteosynthesis. The same catheter was used again for perioperative drug administration, including proton pump inhibitors, corticosteroids, paracetamol, antibiotics, and NSAIDs. |
| **Day 16** | Patient discharged; the catheter was removed after 10 days. |
